# Changes in temperature and precipitation drive shifts in mean flowering timing of tropical plants from 1960 to 2021 across seven locations

**DOI:** 10.1242/bio.062467

**Published:** 2026-03-04

**Authors:** Skylar Graves, Erin A. Manzitto-Tripp

**Affiliations:** ^1^ University of Colorado-Boulder, Ecology and Evolutionary Biology, Boulder, CO 80309, USA; ^2^ University of Colorado-Boulder, Museum of Natural History, Boulder, CO 80309, USA

**Keywords:** Phenology, Climate change, Tropical, Botany

## Abstract

It has been shown that changes in plant flowering times are directly tied to climate change, often being the first and most visible indicators of a broader, ecosystem-wide change. Despite this, tropical latitudes have been markedly understudied. We analyzed 19 tropical species across seven locations from 1960 to 2021. Through a series of Bayesian regression analyses of flowering date on maximum temperature, minimum temperature, and precipitation, we found that flowering dates of tropical plants have changed substantially with changes in climate. Flowering dates have shifted an average of 6.9 days per °C of maximum temperature change, 4.5 days per °C of minimum temperature change, and 0.28 days per mm precipitation. We then computed combined effects of the aforementioned climate variables and found that flowering dates have shifted 15.0 days per unit of combined temperature and precipitation changes (computed as the sum of products of standardized changes in climate variables and their posterior effect estimates). We found no meaningful difference in magnitude of change in flowering of species in consistently hot and wet locations to those in locations with seasonal wet and dry periods (Wilcoxon rank sum, *P*>0.05). Our study demonstrates that tropical ecosystems are not insulated from the impacts of climate change.

## Teaser

**Summary:** This study analyzed the change in flowering date of 19 tropical flowering species using herbarium data, across seven locations from 1960 to 2021, in relation to changes in temperature and precipitation, with findings of an average change in flowering date of 15 days per change in monthly temperature and precipitation.

## INTRODUCTION

Two decades of research have demonstrated that shifts in flowering times of angiosperms have occurred as a result of climate change (Fitter et al., 1995; Amano et al., 2010; Moore and Lauenroth, 2017; Geissler et al., 2023). Responses of plant reproduction to climate change can have cascading impacts across ecosystems (Rudolf, 2019; Zhu et al., 2016; Chen et al., 2020; Shen et al., 2020). For example, climate change has induced changes in insect emergence (Liu et al., 2011; Rafferty and Ives, 2012; Robbirt et al., 2014; Forrest, 2015), which, coupled with changes in flowering timing, can cause pollinator misalignment. Furthermore, climate change can impact migration timing (Cotton, 2003), which in turn can disrupt seed dispersal. These changes can fracture communities and food chains (Morisette et al., 2009; Kharouba et al., 2018; Zhu et al., 2016; Chen et al., 2020). Plant phenology is especially sensitive to a changing climate, with changes in reproductive cycles serving as immediate and visible indicators of broader change and predictors of more drastic ecological responses (Chuine and Régnière, 2017; Brenskelle et al., 2019; Wolkovich and Donahue, 2021). Because plants are the basis of the vast majority of terrestrial ecosystems, understanding changes in plant phenology informs us of broader impacts of climate change.

Studies have conveyed the irreplaceable value of museum specimens in phenological research (Primack et al., 2004; Bertin, 2015; Jones and Daehler, 2018). Nonetheless, the vast majority of research from the prior two decades has emphasized plants in temperate and boreal habitats (Fitter et al., 1995; Amano et al., 2010; Moore and Lauenroth, 2017; Geissler et al., 2023). Tropical systems differ substantially in their consistent warmth and humidity due to proximity to the equator and high biodiversity. In contrast to this now rich body of literature on temperate and boreal plant phenological shifts, very few studies have sought to understand climate change impacts on tropical plants, predominantly focusing on a specific region or on a limited number of taxa (see Reich, 1995; Sakai, 2001; Vrieling et al., 2013; Adamescu et al., 2018; Chapman et al., 2018; Fava et al., 2019; Lima et al., 2021; Davis et al., 2022). The expansion of understanding of tropical flora is critically important to the understanding of the global impacts of climate change. This goal becomes difficult due to the patterns of flowering seen in tropical latitudes, with many species having nearly continuous flowering due to the lack of a cold and dark induced dormancy period (Staggemeier et al., 2020; Davis et al., 2022), meaning they flower through most of the year. Measuring a change in mean flowering date of a species with continuous flowering fails to yield a biologically meaningful result, as a change in mean flowering of a species that flowers year round is unlikely to result in misalignment with pollinators or seed dispersers and therefore have no impact on fitness of the plant or their mutualists (Morisette et al., 2009; Kharouba et al., 2018; Zhu et al., 2016; Chen et al., 2020). Therefore, we must focus our attention on species with discrete flowering times. These are species whose flowering is brief enough to have a measurable change in mean flowering date as well as the possibility to have a misalignment with reproductive mutualists, thus resulting in ecosystem changes.

It is well-established that flowering in plants inhabiting temperate and boreal latitudes is induced primarily by changes in maximum temperature and photoperiod (Primack et al., 2004; Robbirt et al., 2011; Calinger et al., 2013; Rawal et al., 2015). It has also been shown that the use of maximum temperature yields the same results as the use of heating degree days in understanding phenological shifts (Lesica and Kittelson, 2010). It has instead been hypothesized that flowering in tropical plant species is instead triggered in part by minimum temperature (Vrieling et al., 2013; Fava et al., 2019; Lima et al., 2021), which is frequently used as a proxy for night temperatures (Moore and Lauenroth, 2017). Furthermore, tropical locations may have greater annual variation in precipitation compared to annual variation in temperature, thus leading precipitation to be an additional important driver of tropical flowering (Vrieling et al., 2013; Fava et al., 2019; Lima et al., 2021). Tropical climates have two major climatic regimes, those with nearly consistent temperature and precipitation year round, and those with wet and dry seasons (Köppen, 1884a,b; Köppen, 1936; Beck et al., 2018). Due to this, we decided to split our study between these climate regimes to better understand the impacts of climate change on tropical locations.

In the present study, we assembled phenological and climatological data for plants with distinct flowering times inhabiting seasonally dry climates and ever-wet climates (consistent annual temperature and precipitation). Our primary hypothesis is that changes in temperature and precipitation will have an impact on flowering timing in tropical plants. To test this hypothesis, we regressed flowering date on three climate variables: average monthly minimum temperature (°C), average monthly maximum temperature (°C), and total monthly precipitation (mm). Because these climate variables are happening simultaneously, we then calculated the combined impact of changes to all three variables concurrently. The results of our study shed new light on what heretofore has been a largely underexplored topic within global studies of impacts of climate change.

## RESULTS

### Changes in temperature and precipitation as reported by WorldClim

**Fig. 1.**
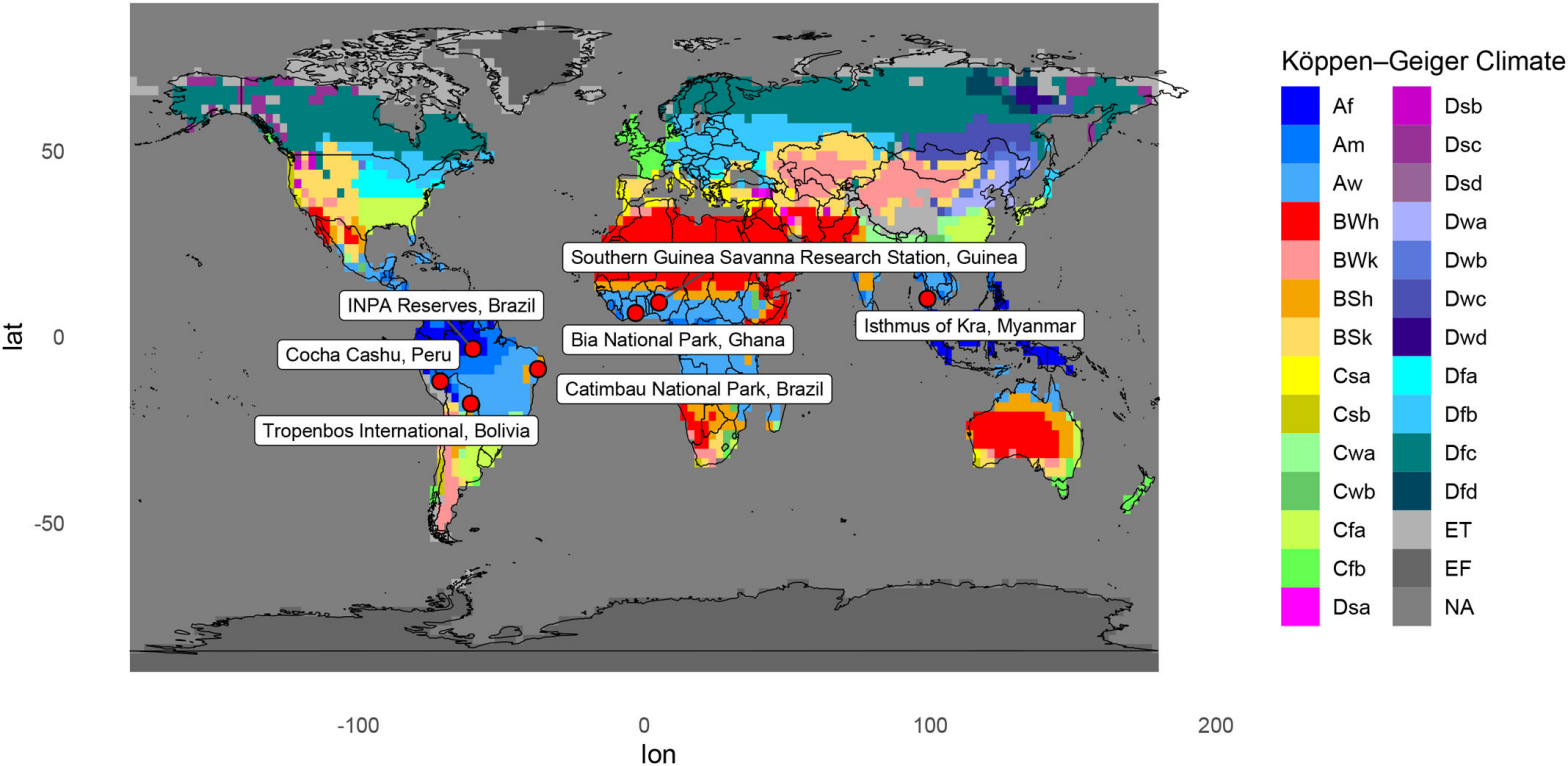
Map of the globe color coded by Köppen climate classification with locations selected for the study marked and labeled.

**Fig. 2.**
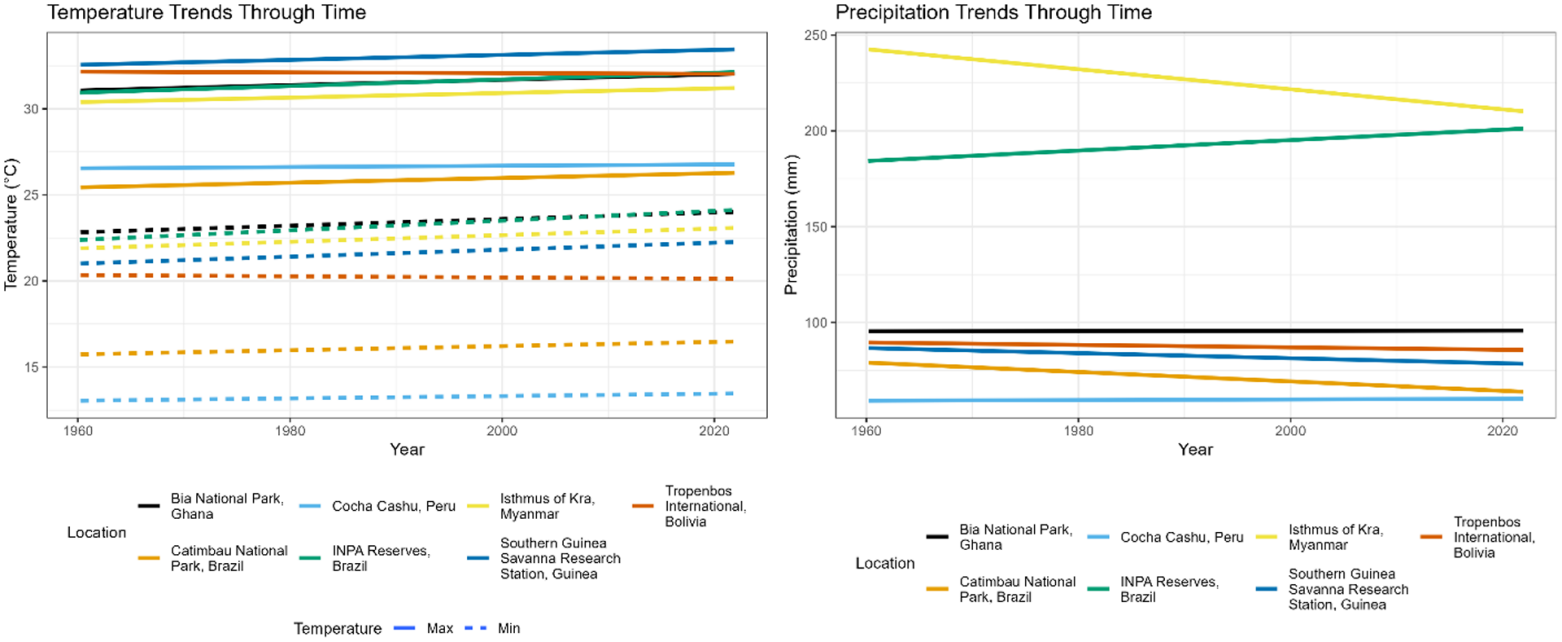
**Change in average monthly maximum temperature (left panel, solid line), change in average monthly minimum temperature (left panel dotted line) and change in total monthly precipitation (right panel) for each location included in the study.** Values acquired by WorldClim and trend lined generated from raw data using geom_smooth.

Across all locations (Table 1; Fig. 1), there was an average increase in maximum temperature of 0.58°C across the 61-year time span, an average increase in minimum temperature of 0.59°C, and an average decrease in precipitation of 14.0 mm (Figs 1, 2). All locations saw an increase in maximum temperature. Bia National Park Ghana had the greatest change, with a 1.00°C increase over 61 years, and Cocha Cashu Peru and Tropenbos International Bolivia both had the smallest change in maximum temperature, with an increase of 0.33°C over 61 years. Five locations showed an increase in minimum temperature, with the greatest increase being 1.58°C in the INPA reserves in Brazil. One location, Cocha Cashu Peru, showed a decrease in minimum temperature, with a cooling of 0.41°C over 61 years. One location, Catimbau National Park, showed no change in minimum temperature. Six locations showed a decrease in precipitation, with the greatest decrease being 69.7 mm at the Isthmus of Kra in Myanmar/Thailand. One location showed an increase in precipitation, INPA Reserves Brazil had an increase of 26.1 mm precipitation over 61 years.

**Table 1.** Approximate latitude and longitude (centroids) of locations included in this study

Location	Latitude	Longitude
INPA Reserves, Brazil	−3.119	−60.021
Tropenbos International, Bolivia	−17.846	−60.738
Cocha Cashu, Peru	−11.888	−71.407
Catimbau National Park, Brazil	−8.592	−37.247
Isthmus of Kra, Myanmar	10.333	99.000
Bia National Park, Ghana	6.504	−3.077
Southern Guinea Savanna Research Station, Guinea	9.296	5.063

### Phenological responsiveness (slope) and change in flowering date

Of the 19 species included in this study, six species have a credibly non-zero slope across all three climate variables as determined by the 95% credible intervals. Five species have a credibly zero slope across all three climate variables, and the remaining eight species have one or more climate variable's slope credibly non-zero and one or more climate variable's slope zero (Table 2).

**Table 2.** Climate type divided by ever wet climates and seasonally dry climates (see Materials and Methods), locations of plant specimens, species name with taxonomic authority

Climate	Location	Species	Max. temp. change in days per °C	Min. temp. change in days per °C	Precip. change in days per mm	Overall change in flowering date per unit climate change	Max. temp. credible non-zero	Min. temp. credible non-zero	Precip. credible non-zero	Number of specimens
Ever-wet	INPA Reserves, Brazil	*Ceiba erianthos* (Cav.) K.Schum.	1.8	−1.3	−0.06	0.55	F	F	T	314
Ever-wet	INPA Reserves, Brazil	*Ceiba jasminodora* (A.St.-Hil.) K.Schum.	1.7	−0.1	0.02	3.4	F	F	F	26
Ever-wet	INPA Reserves, Brazil	*Ceiba schottii Britten & Baker f.*	5.6	−9.9	−0.11	8.01	T	T	T	129
Ever-wet	INPA Reserves, Brazil	*Ceiba trischistandra* (A.Gray) Bakh.	−5.2	−9.6	−0.03	−19.93	F	F	F	38
Ever-wet	INPA Reserves, Brazil	*Porcelia ponderosa* Rusby	4.7	10.9	−0.04	16.61	F	T	F	60
Seasonally dry	Tropenbos International, Bolivia	*Bougainvillea modesta* Heimerl	0.083	−3.7	−0.23	10.23	F	T	T	44
Seasonally dry	Tropenbos International, Bolivia	*Bougainvillea stipitata* Griseb.	−27.7	32.3	0.43	22.88	F	F	F	145
Seasonally dry	Catimbau National Park, Brazil	*Barnebya harleyi* W.R.Anderson & Gates	−14.3	5.6	−0.005	17.92	T	F	F	128
Seasonally dry	Catimbau National Park, Brazil	*Mimosa acutistipula* (Mart.) Benth.	12.3	8.9	0.28	0.18	T	T	T	593
Seasonally dry	Catimbau National Park, Brazil	*Terminalia fagifolia* Mart*.*	15.6	−0.4	−0.097	2.25	F	F	F	560
Seasonally dry	Cocha Cashu, Peru	*Dioscorea bulbifera* L.	13.7	−7.3	0.51	3.89	T	T	T	1029
Ever-wet	Bia National Park, Ghana	*Combretum acutum* M.A.Lawson	20.5	0.09	−0.38	39.89	T	F	T	83
Ever-wet	Bia National Park, Ghana	*Dracaena phrynioides* Hook.	−1.8	6.1	−0.34	7.52	F	T	T	70
Ever-wet	Bia National Park, Ghana	*Stylosanthes erecta* P.Beauv.	−1.2	−4.2	0.26	−11.24	F	T	T	76
Ever-wet	Bia National Park, Ghana	*Terminalia laxiflora* Engl*.*	14.1	−7.3	−0.7	−34.46	T	T	T	183
Ever-wet	Bia National Park, Ghana	*Tetrorchidium didymostemon* (Baill.) Pax & K.Hoffm.	−1.9	0.7	−0.17	−2.49	F	F	F	73
Ever-wet	Bia National Park, Ghana	*Vangueriella nigerica* (Robyns) Verdc.	7.3	0.008	0.79	−1.507	T	F	T	73
Seasonally dry	Southern Guinea Savanna Research Station, Guinea	*Diospyros lotus* L.	−4.6	4.7	0.012	−9.91	T	T	T	661
Seasonally dry	Isthmus of Kra, Myanmar	*Nymphoides aurantiaca* (Dalzell) Kuntze	7.1	−0.7	0.1	21.14	T	T	T	349

Results from Bayesian GLM, regarding change in flowering date in relation to a change in average monthly maximum temperature, average monthly minimum temperature and total monthly precipitation. Results converted to change in flowering date per °C or mm precipitation, respectively. These values were used to calculate a change in flowering date per unit of combined change in temperature and precipitation. Using Posterior values from the regressions, determination of slope being credibly non-zero was computed. T represents True, slope is credibly non-zero. F represents False, slope is not credibly non-zero. Number of specimens used for regression listed. Habit of plant listed.

The use of absolute slope/change in flowering date is used to show magnitude of change regardless of direction of change (earlier or later in the year). Directionality of shift in flowering is discussed separately below. We removed species whose slope was credibly zero for the following averages (Table 3).

**Table 3.** Table includes location, species name and taxonomic authority, IUCN Red List Status, known pollinators, known seed dispersal agents, distribution (as determined by GBIF collections) and habit

Location	Species	IUCN	Pollinator	Seed dispersal	Distribution	Habit
INPA Reserves, Brazil	*Ceiba erianthos (Cav.) K.Schum.*	LC	Bats	Wind/water	Narrowly distributed	Tree
INPA Reserves, Brazil	*Ceiba jasminodora (A.St.-Hil.) K.Schum.*	VU	Moths	Wind/water	Narrowly distributed	Tree
INPA Reserves, Brazil	*Ceiba schottii Britten & Baker f.*	LC	Butterflies	Wind/water	Narrowly distributed	Tree
INPA Reserves, Brazil	*Ceiba trischistandra (A.Gray) Bakh.*	N/A	Bats, bees, hummingbirds	Wind	Narrowly distributed	Tree
INPA Reserves, Brazil	*Porcelia ponderosa Rusby*	LC	Beetles	Animal		Tree
Tropenbos International, Bolivia	*Bougainvillea modesta Heimerl*	LC	Bees, butterflies	Wind/water	Narrowly distributed	Tree
Tropenbos International, Bolivia	*Bougainvillea stipitata Griseb.*	N/A	Butterfly, moth	Wind/water	Narrowly distributed	Tree
Catimbau National Park, Brazil	*Barnebya harleyi W.R.Anderson & Gates*	LC	Bee (*Frieseomelitta meadewaldoi*)	Wind	Widely distributed	Climbing tree
Catimbau National Park, Brazil	*Mimosa acutistipula (Mart.) Benth.*	N/A	Wind, bees	Wind/water	Widely distributed	Tree/shrub
Catimbau National Park, Brazil	*Terminalia fagifolia Mart.*	LC	Bees, butterflies	Cassowaries	Widely distributed	Tree
Cocha Cashu, Peru	*Dioscorea bulbifera L.*	N/A	Insects, wind	Wind – bulbils	Widely distributed	Climbing herbaceous
Bia National Park, Ghana	*Combretum acutum M.A.Lawson*	N/A	Unknown	Wind	Narrowly distributed	Tree
Bia National Park, Ghana	*Dracaena phrynioides Hook.*	N/A	Hawk moth	Primates, birds, rodents	Narrowly distributed	Subshrub
Bia National Park, Ghana	*Stylosanthes erecta P.Beauv.*	N/A	Bees	Water, small animals	Widely distributed	Subshrub
Bia National Park, Ghana	*Terminalia laxiflora Engl.*	LC	Bees	Wind	Widely distributed	Tree
Bia National Park, Ghana	*Tetrorchidium didymostemon (Baill.) Pax & K.Hoffm.*	LC	Insect	Unknown	Widely distributed	Tree
Bia National Park, Ghana	*Vangueriella nigerica (Robyns) Verdc.*	LC	Bees, butterflies, beetles, wind	Wind/water	Narrowly distributed	Tree
Southern Guinea Savanna Research Station, Guinea	*Diospyros lotus L.*	LC	Bees	Birds, mammals	Widely distributed	Tree
Isthmus of Kra, Thailand/Myanmar	*Nymphoides aurantiaca (Dalzell) Kuntze*	LC	Insect	Water	Widely distributed	Aquatic

### Comparison based on climate

**Fig. 3.**
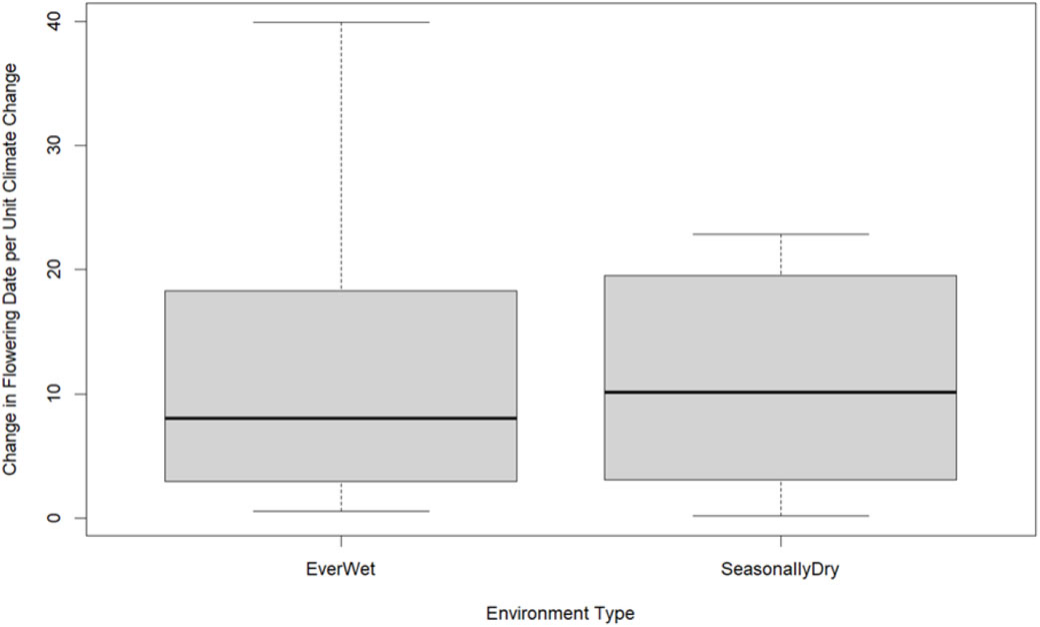
Box and whisker plot showcasing average and range of change in flowering date between differential tropical climate regimes.

Of the 14 species included in our study that have a credibly non-zero slope, eight occurred in two locations that were classified as ever-wet. Wilcoxon rank sum test between ever-wet and seasonally dry climate results indicated that there was not a significant difference in change in flowering date based on climate change occurring in ever-wet versus seasonally dry environments (W=60, *P*-value=0.206) (Fig. 3).

### Directionality of shift

**Fig. 4.**
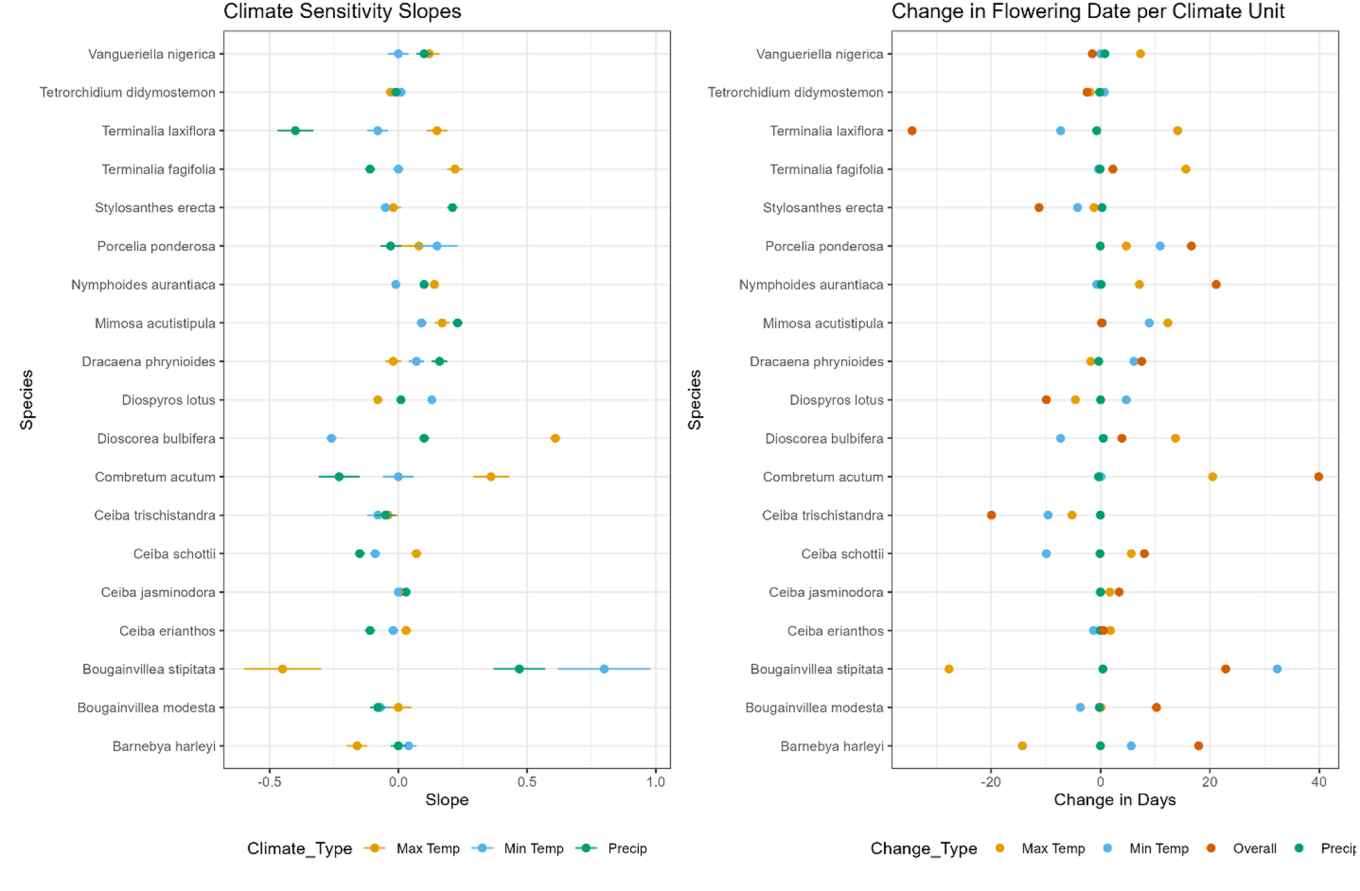
**Effect of climate on flowering date.** (Left) Slope of regression representing change in flowering date in relation to change in average monthly maximum temperature (yellow), average monthly minimum temperature (blue) and total monthly precipitation (green) for each species, with 95% confidence interval error bars. (Right) Change in flowering date per change in °C of maximum temperature (yellow), minimum temperature (blue) and mm precipitation (green) and overall change in the combined effects of all three aforementioned variables (red).

Out of the 14 species in the study that have a credibly non-zero slope, four out of 14 (28.5%) species had a negative change in flowering date due to combined effects of climate change, indicating flowering is now occurring earlier in the year as compared to historically. In contrast, the majority of species [10 out of 14 (71.5%)] underwent a positive change in flowering date due to the combined effects of climate change, indicating flowering is now occurring later in the year compared to historical records (Fig. 4; Table 2). Three of the four species flowering earlier in the year are in ever-wet climates and one species is in a seasonally dry climate. The average change in flowering date per unit combined climate change for these four species is 14.3 days. Of the ten species flowering later in the year, five are in ever-wet climates and five are in seasonally dry climates. The average change in flowering date per unit combined climate change for these ten species is 12.6 days.

## DISCUSSION

Our results indicate that tropical plant species with discrete flowering periods experience measurable shifts in flowering timing due to climate change. We found a wide range of responses to climate change across the species in this study, ranging from a change in flowering date of 0.18 days to 39 days per unit of combined temperature and precipitation changes and resulting in an average change in flowering date of 15.1 days between 1960–2021 (∼2.4 days per decade).

We recognize the small number of taxa used here, limited by the availability of species with short flowering periods and regular collections throughout time, is not representative of the biodiversity seen in these locations, or the global tropics. The main benefit of this study is the ability to quantify the changes in flowering date seen in species with discrete flowering times, whose change in flowering date is measurable and biologically meaningful (as opposed to species with continuous flowering throughout the year). Species with discrete flowering periods are at higher risk of misalignment with their mutualists, leading to a decrease in fitness. Measuring these changes in flowering in relation to climatic variables gives us the ability to link that change in flowering to climate variables, which in turn allows us to connect these changes to those observed in pollinators and seed dispersers. By focusing on these species, we are able to track the impacts of flowering on both the most measurable and most vulnerable plant species in these tropical locations.

### Changes in temperature and precipitation drive tropical plant flowering shifts

We investigated flowering plant responses to climate change through a lens of changes in temperature and precipitation. Of the species with a credibly non-zero slope, we documented an average change in flowering date of 15.1 days per unit of combined temperature and precipitation change over 61 years. Similar studies, albeit predominantly in non-tropical habitats, have demonstrated plants have undergone 2–10 days of phenological change in flowering date per 1°C (Primack et al., 2004; Robbirt et al., 2011; Panchen et al., 2012; Park and Schwartz, 2015), although a smaller number of studies have shown larger changes in flowering dates, up to 27 days per 1°C (Fitter et al., 1995; Gaira et al., 2011; Calinger et al., 2013; Rawal et al., 2015).

In our dataset, when exploring change in flowering date in relation to maximum temperature, five species occur in the 2–10 days per 1°C frame of change, with the remaining species split between more than 10 days per 1°C and fewer than 2 days per 1°C. Regarding minimum temperature, ten species shifted their flowering date between 2–10 days per 1°C and four species shifted fewer than 2 days per 1°C. The variation in change in flowering date in relation to change in temperature across the 14 species in our study is greater than what is typically seen in the literature, albeit predominantly in non-tropical habitats, with a substantial portion of species showing a smaller change in flowering date than the established 2–10 days per 1°C, as well as a substantial portion of species showing a greater change in flowering date. Temperate species are likely driven by temperature and photoperiod, whereas this variation seen in tropical species may be due to the lack of annual temperature variation, or cold induced dormancy. We are aware that the limited number of species in this study is not representative of these locations or the global tropics and highly encourage more studies on the relationship between change in temperature and flowering date in tropical regions across greater taxa.

It is difficult to compare our results regarding changes in tropical plant phenology in relation to changes in precipitation, as this is largely under-studied in the literature. It has been shown that an increase in precipitation has resulted in a delay in spring flowering in semi-arid grasslands and steppes (Lesica and Kittelson, 2010; Von Holle et al., 2010; Mazer et al., 2013; Matthews and Mazer, 2016; Zhou et al., 2019). This may be due in part to the increase in precipitation, which decreases soil temperature (Lesica and Kittelson, 2010; Matthews and Mazer, 2016; Zhou et al., 2019). In tropical latitudes intra-annual variation in precipitation may be greater than variation in temperature and therefore may have a greater impact on flowering as compared to temperate latitudes. Only one study has linked the change in flowering date per change in unit precipitation and that investigation showed an advance in flowering date of 0.12 days per decrease of 1 mm of precipitation (Lesica and Kittelson, 2010). This number is less than half that demonstrated in the present study, which documents an average change of 0.28 days per mm precipitation change. Given that anthropogenic climate change is driving shifts in precipitation globally, it would prove fruitful to study the impacts of changes in precipitation on flowering across various biomes globally, in both arid and humid climate types, as well as across latitudes and altitudes.

#### Large range in variation of tropical plant flowering shifts

The variation in flowering response seen across species in this study is quite substantial. In our study, this variation exists within a single lineage at a single location. For example, in the INPA reserves, we documented 0.5 days of change per unit climate change in *Ceiba erianthos* versus 19.9 days of change per unit climate change in *Ceiba trischistandra* (Table 4)*.* Distribution does not appear to have an impact on severity of shift in flowering time, as great variation in change in flowering date was seen across both narrowly and widely distributed species. Narrowly distributed species had an average change in flowering date of 11.9 days per unit combined climate change, whereas the more widely distributed species had an average change in flowering date of 12.6 days per unit combined climate change. A widely distributed species may be less at risk of extinction due to changes in fitness at a single location (as compared to narrowly endemic species), changes in fitness of any species will still have cascade impacts on all other species that rely on them. Prior studies have shown that other factors, such as growth form, could impact and help to explain observed variation. For example, Geissler et al. (2023) showed that correlation between warming and flowering timing is stronger in woody plants than herbaceous plants. In our study, however, growth form fails to explain observed variation given all but two species in this study are woody (Table 2). Further studies that investigate the effects of functional traits of plants on flowering time will likely yield insights and should be prioritized. Furthermore, future studies should pay particular attention to variation in phenology seen across species in a single location, with an aim to determine drivers of this variation.

**Table 4.** Averages of change in absolute slope and days per °C or mm precipitation, as well as change in combined effect of temperature and precipitation

	Max. slope	Min. slope	Precip. slope	Max. days per °C	Min. days per °C	Precip. days per mm	Overall change per unit climate change	Number of species
Average (All)	0.107	0.06	0.15	6.9107	4.5698	0.2818	15.08819	14
Ever-wet	0.10625	0.0575	0.17375	7.16275	5.023375	0.33825	14.97825	8
Seasonally dry	0.193333	0.1	0.086667	8.722833	5.185667	0.191833	10.54986	6

Average shown for all species with credibly non-zero slope, as well as averages separated by ever-wet and seasonally dry climates. Number of species included.

#### Directionality of flowering shifts

Out of the 14 species included in our study with credibly non-zero slopes, 28.5% are now flowering earlier (combined effects of temperature and precipitation) and 71.5% are flowering later in the year. This variation is within a similar range to those seen at temperate locations (Williamson et al., 2025). Due to the small sample size, it is difficult to generalize whether ever-wet locations are more affected by climate change than seasonally dry ones.

#### Differences in change in flowering date by climate type

We observed an average change in flowering date in ever-wet locations of 14.9 days per unit of combined climate change and a change in flowering date in seasonally dry locations of 10.5 days per unit of combined climate change. The difference of 4.4 days per unit of combined climate change is moderate. This variation is shown across eight species in two ever-wet locations and six species in five seasonally dry locations. Due to the small sample size, it is impossible to determine if ever-wet locations are moderately more affected by climate change than seasonally dry ones across more than the species shown here.

### Conclusion

Our work lays a foundation for the study of tropical flowering phenology in relation to climate change. By analyzing the impacts of temperature and precipitation on flowering time, we have sought to interpret the impacts of climate change on plants inhabiting understudied and vulnerable tropical latitudes. Despite contrasting patterns of temperature and precipitation in ever-wet versus seasonally dry ecosystems, we determined that plants inhabiting these two ecosystems have responded comparably to the impacts of climate change and therefore both require attention from a conservation standpoint. However, more work is needed to understand the impacts of climate change on tropical floras worldwide, especially work that expands taxon sampling and/or work that analyzes the impacts that biotic factors such as pollinators have on flowering timing. The collection and digitization of herbarium specimens of more tropical plant species will enable new study of the impacts of plant functional traits on changes in flowering timing. Full-scale harnessing of the power of herbarium specimens will thus facilitate the assessment of phenological changes based on myriad factors, such as habit, pollination syndrome, native status, and more. A resolution to study plants from overlooked tropical latitudes is vitally necessary to fully understand the impact of climate change on our globe and adequately prepare for the consequences.

## MATERIALS AND METHODS

Determining the geographical boundaries of our study sites involved two steps. First we determined the different climate regimes of the tropics using the Köppen classification (Köppen, 1884a,b; Köppen, 1936; Beck et al., 2018). The Köppen classification describes four climate types globally. To facilitate downstream work, we grouped the Af (rainforest) and Am (monsoon) regions into a single group, here termed ever-wet climates. We then grouped Aw (savanna, dry winter) and As (savanna, dry summer) regions together into a single region, here termed seasonally dry climates.

Seasonally dry tropical climates have distinct wet and dry seasons, with rainfall of 50 cm in the dry season(s) to 175 cm in the wet season(s). Precipitation occurs most frequently as a result of convectional thunderstorm activity. Seasonally dry tropical climates have a larger temperature range than ever-wet ecosystems, ranging from 19–20°C in winter and 24–27°C in the summer. In contrast, ever-wet climates have heavy precipitation, around 150–1000 cm and consistent, year-round high temperatures (∼30°C) with little intra-annual variation (Köppen, 1884a,b; Köppen, 1936; Beck, 2018; Cui et al., 2021).

Second, following the climate-study site designations, we sought out biological research stations or preserves to utilize as representative locations from which to derive phenological data. Research stations are well-understood to typically have increased rates of herbarium specimen collection in tropical habitats compared to other areas. This increase in the frequency of collection efforts over time in turn helps minimize collector bias (Primack et al., 2004; Jones and Daehler, 2018; GBIF, 2025a,b,c,d,e,f,g,h,i,j,k,l,m,n,o,p,q,r,s,t,u,v,w,x,y,z,aa,bb,cc,dd,ee,ff,gg,hh,ii,jj,kk) while simultaneously yielding a sufficient numbers of species with regular specimen collections through time to effectively visualize changes. Locations (and subsequent species) were checked for regularity of herbarium specimen collections from January 1960 to December 2021, to ensure the ability to measure changes in flowering across time. This selection process resulted in ten locations that were subsequently pared down to seven due to species' data availability. Of the seven locations, four were in the Neotropics, two were in the Afrotropics and one was in southeast Asia. Furthermore, two of these locations are in ever-wet climates and five are in seasonally dry climates (Table 1, Fig. 1).

### Data retrieval and dataset compilation

#### Construction of flowering datasets

Studies have conveyed the irreplaceable value of museum specimens in phenological research, with multiple studies showcasing the use of herbarium specimens in the study of flowering phenology yield comparable results to that of field studies, while providing a greater geographic and temporal scale than is possible from field studies (Primack et al., 2004; Bertin, 2015; Jones and Daehler, 2018). Standard practice for the preparation of an herbarium specimen is the inclusion of both vegetative tissue as well as flower, fruit or ideally both. This preparation makes herbarium specimens ideal for the study of reproductive plant phenology.

Flowering data were derived specifically from museum specimen databases available via GBIF (GBIF.org, accessed January 23rd- April 17th, 2025a,b,c,d,e,f,g,h,i,j,k,l,m,n,o,p,q,r,s,t,u,v,w,x,y,z,aa,bb,cc,dd,ee,ff,gg,hh,ii,jj,kk). The GBIF database contains both museum specimen data and observation records. We used only museum specimen data. A vast majority of herbaria globally upload all their digitized specimens to GBIF as well as other online databases. Due to this, GBIF is an ideal resource for acquiring museum specimens from a multitude of scientific institutions. Using the map polygon feature on GBIF, a polygon was drawn around the boarders of each preserve. A list of all angiosperm species within the bounds of said polygons was compiled (GBIF.org, January 23rd- April 17th, 2025a,b,c,d,e,f,g,h,i,j,k,l,m,n,o,p,q,r,s,t,u,v,w,x,y,z,aa,bb,cc,dd,ee,ff,gg,hh,ii,jj,kk). We manually reviewed photos of digitized museum specimens to determine presence or absence of flowering. All flowering phenophases were included, i.e. flowering was considered ‘present’ the gamete-producing floral whorls (i.e. the androecium and/or gynoecium) were visible.

It has been shown that the unwanted impacts of collector bias can be ameliorated with large datasets and utilization of mean flowering, as opposed to peak or first flowering (Jones and Daehler, 2018). First flowering refers to the time when the earliest flowers open, whereas peak flowering refers to the time when the largest number of flowers are in bloom. Due to the nature of museum specimens, the measure of peak or first flowering cannot be guaranteed, thus we have decided to use mean flowering, as mean flowering or average flowering has been shown to be accurately represented by museum specimens as well as be biologically informative (Davis et al., 2015; Jones and Daehler, 2018). Using the filter by month feature on GBIF, we recorded every month in which flowering was occurring, for each species, in each year, across all collections. This study is a continuation of a previous study in which we measured change in flowering date across time. In that study we analyzed the statistical differences in a variety of specimen inclusion criteria, including duration of flowering period, minimum specimens and length of date range. From that analysis we constructed the following criteria that include the largest number of species while maintaining the efficacy of measuring change in flowering date. We have provided these analyses in the open access data available on GitHub. Species were required to meet the following criteria for inclusion: species flower for four consecutive months or fewer and had a minimum of 20 preserved museum specimens between 1960 and 2021. Utilizing only species that flower once a year for a brief period reduced the breadth of taxa considerably; however, we feel utilizing species whose flowering duration is brief allows for a meaningful measure of change in flowering date. This trade off inhibits the ability to make location-wide conclusions about the impacts of climate change; however, it allows for greater accuracy in the analysis of the species included. When analyzing species with continuous flowering we found mean flowering date changes had high uncertainty and poor fit, with results multiple orders of magnitude greater than expected values. That is to say, species whose flowering period spans the year fail to yield valid and biologically meaningful inferences in the change in flowering across time. Application of these species reduction criteria resulted in a reduction of the total number of species to be analyzed to 19 with an average of 252 specimens per species (Table 2). Specimens that met the above criteria were downloaded from GBIF in DarwinCore format. Replicate collections from the same individual on the same date were pruned from the dataset. Species name, Julian collection date, and year were retrieved for analysis.

Of the species included in this study, 17 were trees, three were shrubs or subshrubs, two were climbing, one was an herb, and one was aquatic (Table 3). All species included in this study utilize animal pollinators, with most using a variety of insects and some using vertebrates such as bats and birds. Three species also utilize wind pollination. Of the species included in this study, 16 species use abiotic seed dispersal, such as wind and water, and five use animal seed dispersal. Using GBIF to estimate distribution we found that nine species were more narrowly distributed, and the remaining ten species were more widely distributed. According to the IUCN red list, 12 species are categorized as Least Concern and One species is categorized as Vulnerable (*Ceiba jasminodora*). Of the species included in this study, the IUCN did not have a report on eight species. The IUCN reports the population is stable for 13 species. All other population changes are Unknown (Table 3).

#### Construction of climate datasets

Data from WorldClim historic monthly climate data was acquired. These data were downscaled from CRU-TS-4.06 by the Climatic Research Unit (Fick and Hijmans, 2017; Harris et al., 2020), University of East Anglia, UK, using WorldClim 2.1 for bias correction. The available data were average minimum temperature (°C), average maximum temperature (°C), and total precipitation (mm) for each month from January 1960 to December 2021. The spatial resolution for the selected data was 2.5 min (∼21 km^2^ at the equator). This resolution is the narrowest resolution available for this type of climate data, limiting the specificity of climate parameters. Rasters were stacked and monthly average minimum temperature (°C), average maximum temperature (°C), and total precipitation (mm) were extracted for each location based on the centroid latitude and longitude coordinates for each research station included in the study (Table 1). Values were concatenated by location.

### Statistical analyses

These data have a circular distribution owing to some species flowering period crossing the new year. To model seasonality appropriately, flowering dates were transformed from Julian day to radians to represent circular time (Staggemeier et al., 2020; Davis et al., 2022; Williamson et al., 2025). Radians were centered around day 183 (July 2) to align midyear with the origin of the circular scale, using the transformation:

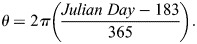
The resulting angle was bounded between −π and π. All climate variables were standardized (mean=0, s.d.=1) prior to analysis.

To test the separate impacts of each climate variable, we modeled flowering date as a function of monthly maximum temperature (°C), minimum temperature (°C), and total precipitation (mm) using the brms package in R (Bürkner, 2017; R Core Team, 2025). Prior to modeling, each climate variable was standardized (mean-centered and scaled to unit variance) to facilitate coefficient comparison. We specified priors for all regression parameters: Normal (0, 0.1) for slopes, Normal (0, π) for the intercept, and Gamma (10, 1) for the von Mises concentration parameter (Staggemeier et al., 2020; Davis et al., 2022; Williamson et al., 2025). We used a Hamiltonian Monte Carlo estimation with a max tree depth of 15 and a No U-Turns Sampler with a target acceptance rate of 0.999. Posterior draws were transformed from radians to days by scaling regression coefficients:

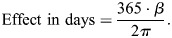
To contextualize flowering responses in terms of recent climate change, we calculated the mean climate conditions for the earliest and latest years in the dataset. The difference in mean values for monthly maximum temperature (°C), minimum temperature (°C), and total precipitation (mm) between values centered on start and end years was expressed in standard deviation (s.d.) units.

To estimate total change in flowering time attributable to observed climate trends, we multiplied the s.d.-unit change in each climate variable by its respective posterior slope (in days per s.d.). Finally, we converted these to raw units (°C or mm) using the standard deviations of the unscaled climate variables and reported flowering date shifts in days per unit change.

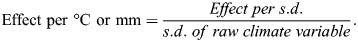
Because all three climatic variables used here are changing simultaneously (Vleminckx et al., 2024), we calculated a ‘combined climatic effect’ value that simultaneously takes into account the impacts of changes in maximum temperature, minimum temperature and total precipitation (Vleminckx et al., 2024). The combined estimated flowering date change (in days) due to climate change was computed as the sum of products of standardized changes in climate variables and their posterior effect estimates. We computed 95% credible intervals from our Bayesian analyses to determine if the slope is credibly non-zero. This is done for each species and each climate variable separately.

To test for potential differences in observed change in flowering (measured in days per unit climate change) across climate types, we conducted a Wilcoxon rank sum exact test, comparing the ‘combined climate effect’ values for species found in ever-wet and seasonally dry climates. Wilcoxon rank sum exact test was chosen due to the non-normality in the data.
